# A case of Barrett’s esophageal cancer with gastric mucosa-associated lymphoma

**DOI:** 10.1186/s40792-020-00956-0

**Published:** 2020-08-06

**Authors:** Fumiaki Shiratori, Isamu Hoshino, Hisashi Gunji, Nobuhiro Takiguchi, Yoshihiro Nabeya, Hideaki Shimada

**Affiliations:** 1 Division of Gastroenterological Surgery, Chiba Cancer Center, 666-2 Nitona-cho, Chuo-ku, Chiba, 260-8717 Japan; 2 Department of Surgery, Toho University Medical Center, Omori Hospital, Graduate School of Medicine, Toho University, 6-11-1 Omori-Nishi, Ota-ku, Tokyo, 1438541 Japan; 3 Department of Clinical Oncology, Toho University Graduate School of Medicine, 6-11-1 Omori-nishi, Ota-ku, Tokyo, 1438541 Japan

**Keywords:** Esophageal cancer, Gastric mucosa-associated lymphoma, Surgery, Gastric conduit

## Abstract

**Background:**

Although the first-line therapy for early-stage gastric mucosa-associated lymphoid tissue lymphoma is the eradication of *Helicobacter pylori*, the effect of eradication in *Helicobacter pylori*-negative cases is unclear. In this case report, we describe a surgical option for a case of Barrett’s esophageal cancer with concurrent gastric mucosa-associated lymphoid tissue lymphoma.

**Case presentation:**

A 79-year-old man was admitted to our hospital with Barrett’s esophageal cancer and gastric mucosa-associated lymphoid tissue lymphoma. Initially, we performed endoscopic submucosal dissection for Barrett’s esophageal cancer. Since residual tumor was observed after the endoscopic submucosal dissection, we performed an esophagectomy with two-field lymph node dissection, which was followed by placement of a gastric conduit via the posterior mediastinal route. He was discharged 14 days after surgery. Although no additional treatment exists for mucosa-associated lymphoid tissue lymphoma, no recurrent disease has been detected to date.

**Conclusion:**

An option to use a portion of the stomach with low-grade malignant mucosa-associated lymphoid tissue lymphoma as a conduit after esophagectomy was suggested.

## Background

Barrett’s esophagus is present in 10–20% of patients with gastroesophageal reflux disease and 2–7% of the general population, with an incidence between 23.1 and 32.7 per 100,000 individuals [1]. Mucosa-associated lymphoid tissue (MALT) lymphoma is the most common type of extranodal non-Hodgkin lymphoma and primarily involves the stomach. Patients with gastric MALT lymphoma can be asymptomatic or may present with vague complaints of dyspepsia. Such a malignancy is associated with autoimmune disorders or chronic inflammation, which in most cases is caused by *Helicobacter pylori* (Hp) infection. Gastric MALT lymphoma usually manifests as a low-grade lymphoma, and in a minority of cases, low-grade disease transitions into a high-grade malignancy. The eradication of Hp infection with standard therapy leads to complete remission of the lymphoma in approximately 80% of cases [2]. On the contrary, no clear therapeutic consensus has been established for Hp-negative low-grade malignant MALT lymphoma [3].

Subtotal esophagectomy followed by reconstruction using a gastric conduit is a standard procedure in patients with esophageal cancer [4]. Another surgical option for esophageal cancer patients who also have gastric cancer or a remnant stomach is reconstruction using the colon or jejunum. Colonic or jejunal reconstruction is a high-risk surgical procedure that is associated with severe surgical stress [5]. For the patient with Barrett’s esophageal cancer presented here, we used the stomach with MALT lymphoma as the organ for reconstruction of the esophagus.

## Case presentation

**Fig. 1:**
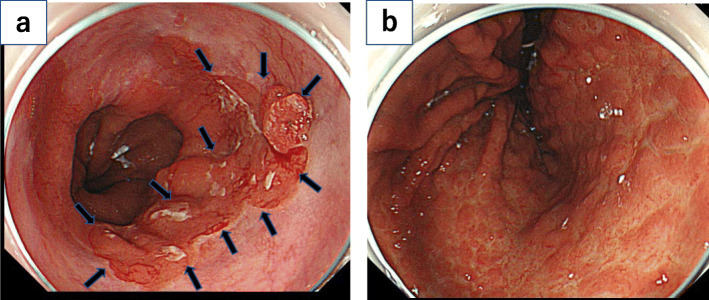
Diagnosis of esophageal cancer based on upper gastrointestinal endoscopy findings. **a** Upper gastrointestinal endoscopy showed a I + IIa lesion approximately 40 mm in size that formed a semicircular pattern around the posterior wall of the esophagus 37 mm from the incisors. **b** Brownish mucosa was frequently observed from the lower body to the upper body of the stomach

**Fig. 2:**
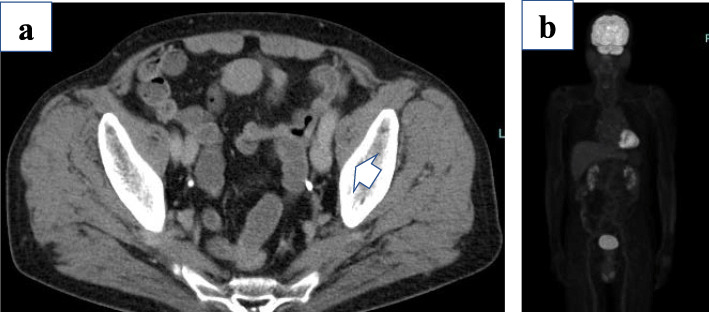
CT and PET-CT findings. **a** CT revealed an approximately 30-mm enlarged lymph node in the mesentery of the small intestine. **b** PET showed no FDG uptake

**Fig. 3:**
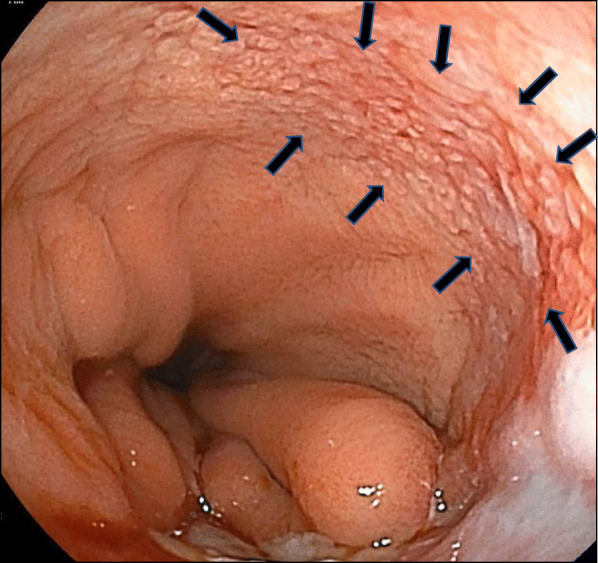
Upper gastrointestinal endoscopy findings after ESD. Upper gastrointestinal endoscopy showed a rough mucous membrane that was located approximately 30 mm below the ESD scar

A 79-year-old Japanese man was referred to our hospital for esophageal cancer. Gastrointestinal endoscopy revealed a 37-cm tumor starting at the incisors with Barrett’s esophagus (Fig. 1a) and multiple brownish mucous membranes from the lower to the upper body of the stomach (Fig. 1b). A biopsy of the esophageal tumor revealed a well-differentiated adenocarcinoma, whereas biopsy of the brownish mucous membranes in the stomach revealed MALT lymphoma. A large lymph node in the mesenteric membrane of the small intestine (Fig. 2a) was confirmed by a computed tomography scan. However, no accumulation of contrast agent was observed in the lymph node on positron emission tomography (PET) (Fig. 2b). All laboratory data were within the normal range: the carcinoembryonic antigen level was 2.7 ng/mL (normal range < 5.0 mg/dL), the cytokeratin 19 fragment level was 1.3 ng/mL (normal range < 3.5 mg/dL), the squamous cell carcinoma-associated antigen level was 1.4 ng/mL (normal range < 1.5 ng/dL), and anti-Hp antibody testing was negative. According to the Union for International Cancer Control Tumor–Node–Metastasis classification (8th edition), Barrett’s esophageal cancer was classified as cStage IA (cT1bN0M0). We elected to perform endoscopic submucosal dissection (ESD) as a first-line treatment option considering the balance of surgical risk and the presence of MALT. The pathological analysis of the ESD specimen revealed a well-differentiated adenocarcinoma pDMM, ly (+), v (−), HMX, VM0. Furthermore, gastrointestinal endoscopy revealed the presence of remnant tumor after the ESD (Fig. 3). Therefore, we elected to perform radical surgery for Barrett’s esophageal cancer only as a second option.

### Surgical procedures

Esophagectomy with two-field lymph node dissection and gastric conduit reconstruction via the posterior mediastinal route were performed. Since a large lymph node was located in the mesentery of the small intestine, both the lymph node and the small intestine were resected together. The operation time was 6 h and 18 min, with an estimated blood loss of 80 mL.

### Pathological findings

**Fig. 4:**
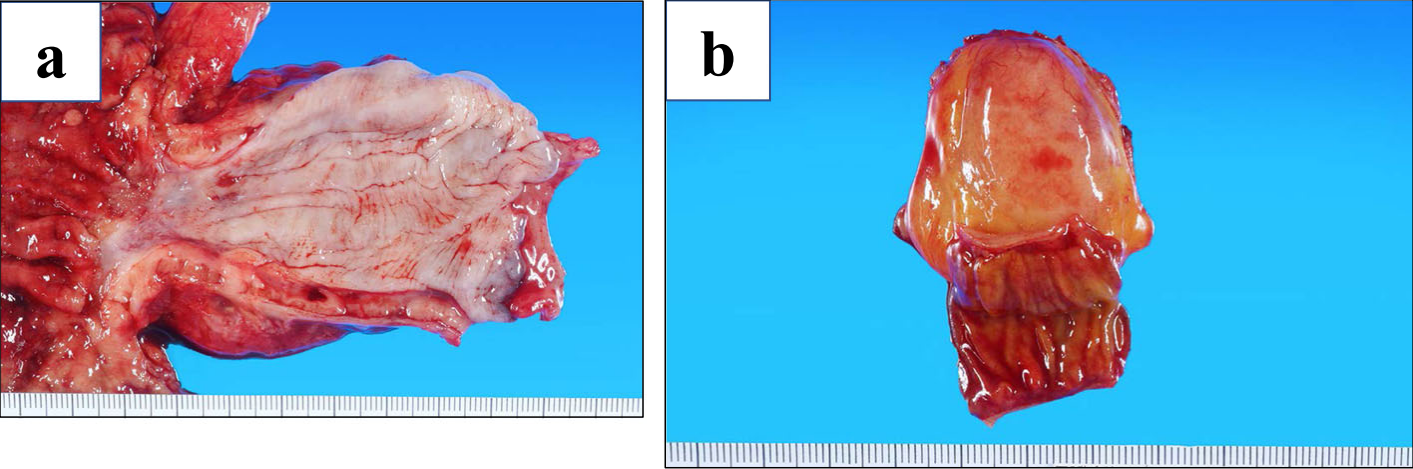
Macroscopic findings of the resected specimen. **a** The resected tumor measured 30 × 20 mm, with a post-ESD scar visible on the lower esophagus. **b** Intramesenteric lymph node resected with the small intestine

The resected tumor measured 30 × 20 mm in short-segment Barrett’s esophagus and contained a post-ESD scar (Fig. 4a). Pathologic analysis showed a well-differentiated adenocarcinoma with short-segment Barrett’s esophagus, 0-IIc, 30 × 20 mm, pT1a-SMM ly0 v0 N0 M0, and pStage0 according to the 8th edition of the UICC TNM staging system. This patient was diagnosed with MALT lymphoma of the cell component in the large abdominal lymph node (Fig. 4b). The MALT lymphoma did not invade the esophagus, but rather, it spread throughout the stomach, including the resected margin.

### Postoperative course

The patient’s postoperative course was uneventful. The patient resumed eating on postoperative day 7 and was discharged from the hospital on postoperative day 14. No additional treatment was administered for MALT. Thus far, with respect to the MALT lymphoma, no recurrent disease except that in the gastric tube has been observed.

### Discussion

According to the European Society of Medical Oncology guidelines for gastric MALT lymphoma [6], the patient was classified as stage I based on the Lugano staging system. The patient was negative for anti-Hp antibody, and thus, we prioritized treatment for Barrett’s esophageal cancer over treatment for the MALT lymphoma. According to the “Practical Guidelines for Hematological Malignancies (2018) of the Japanese Society of Hematology,” radiotherapy is recommended as the initial treatment in patients with Hp-negative gastric MALT lymphoma based on category 2B evidence. Antibiotic treatment was also recommended in a previous report [3]. Since these evidence levels were low and because substantial time is required to obtain a therapeutic effect regardless of whether radiation therapy or antibiotic therapy is applied, we decided that esophageal cancer treatment should proceed.

Although FDG uptake during PET-CT is frequently observed in many MALT lymphomas with high-grade malignant potential [7], this case did not show abnormal FDG uptake in the mesenteric lymph nodes or in other lesions. We removed the enlarged lymph nodes during the surgery for esophageal cancer and submitted the specimens for pathological examination. After consultation with a hematologist, we elected to prioritize esophageal cancer treatment.

Initially, we decided to perform ESD because Yu et al. reported that elderly patients who underwent esophagectomy had significantly lower cancer-related and 5-year overall survival (OS) rates than relatively younger patients [8]. However, pathological findings of ESD specimens revealed lymph node infiltration with possible positive horizontal margins. Moreover, post-ESD gastrointestinal endoscopy performed during follow-up detected residual tumor. Since Barrett’s esophageal cancer showed scar tissue-related stenosis after ESD, we could not perform additional ESD.

We believe that chemoradiation therapy may likely worsen the stenosis. Therefore, we considered surgery to be the best option in this case. We elected to perform radical esophagectomy as an additional treatment. For this elderly patient, it was important to determine which organ should be used for reconstruction after esophagectomy. Reconstruction using the colon or jejunum should be performed after total gastrectomy [9]. Actually, colonic or jejunal reconstruction may be associated with higher morbidity and mortality rates than a gastric conduit [10–13]. Shimada et al. [5] reported that the use of a colon substitution in patients with remnant stomach was a significant independent risk factor for poor 5-year OS rates compared with gastric substitution. No reports have been published on residual MALT lymphoma at the resected margin of the stomach. Therefore, the prognosis of MALT lymphoma that remains on the gastric tube after esophagectomy is unknown. The patient experienced no complications and was discharged from the hospital on postoperative day 14.

Initially, we considered the MALT lymphoma to be a localized lesion in the stomach, but a pathologic analysis revealed that the lymphoma in the mesentery of the small intestine was actually metastases of the MALT lymphoma. In many cases, the MALT lymphoma is less likely to have a worse prognosis even if residual lymphoma cells are present [14]. Radiation therapy and chemotherapy have been reported to be treatments for MALT lymphoma, but a precise treatment has not yet been established. In this case, the swollen lymph nodes in the mesentery were removed during surgery, and no more obvious metastatic lymph nodes remained. Therefore, considering the patient’s age, the patient was followed-up without additional therapy.

Thieblemont et al. published data on a new prognostic factor for MALT lymphoma. Three individual factors with the greatest prognostic significance were age > 70 years, Ann Arbor stage III or IV, and an elevated lactate dehydrogenase (LDH) level. These factors allowed the three groups to be classified as follows: low, intermediate, and high risk (according to the presence of 0, 1, or > 2 of these factors, respectively). The 5-year OS rates in the low-, intermediate-, and high-risk groups were 98.7%, 93.1%, and 64.3%, respectively [15]. Since this case was classified as intermediate risk, the 5-year OS rates were estimated to be over 90%, which is considered to be much better than the OS rates estimated as a result of his esophageal cancer. In this case, we decided not to administer additional treatment for the MALT lymphoma and only intended to perform observations during follow-up. At 6 months after surgery, no recurrent esophageal cancer or MALT lymphoma was observed. Although the observation period was short, upfront esophageal cancer surgery without any postoperative complications was the preferred approach for this patient.

To our knowledge, based on a search for literature published between January 1983 and March 2020 in the Japan Medical Abstracts Society and PubMed databases using the key words *esophageal cancer*, *gastric MALT lymphoma*, and *surgery*, this is the first report of esophageal cancer with gastric MALT lymphoma. We believe that it is possible to use the stomach despite the presence of low-grade MALT lymphoma as a reconstructed esophagus in elderly patients. If the stomach with MALT lymphoma can be used as a reconstructed organ, the choice of surgery may be expanded, and postoperative complications can be reduced.

## Conclusions

Here, we present a surgical option for an elderly patient with Barrett’s esophageal cancer combined with gastric MALT lymphoma. Considering the balance of surgical risk and malignant potential, we used a gastric conduit containing MALT lymphoma.

## Data Availability

Data sharing is not applicable to this article, as no datasets were generated or analyzed during the study.

## Abbreviations

MALT lymphoma: Mucosa-associated lymphoid tissue lymphoma
ESD: Endoscopic submucosal dissection
OS: Overall survival
